# Supplementary material for: SLEEPY: a comprehensive Python module for simulating relaxation and dynamics in nuclear magnetic resonance
Source: Nat Commun. 2025 Oct 20;16:9278. doi: 10.1038/s41467-025-65091-6 (PMC12537869; doi:10.1038/s41467-025-65091-6)
Supplement: Supplementary file 1 — Description of Additional Supplementary Files [file 41467_2025_65091_MOESM1_ESM.docx]

**Description of Additional Supplementary Files**

**Supplementary Software 1:** Python code used for benchmarking SLEEPY
